# *Microviridae* Goes Temperate: Microvirus-Related Proviruses Reside in the Genomes of *Bacteroidetes*

**DOI:** 10.1371/journal.pone.0019893

**Published:** 2011-05-10

**Authors:** Mart Krupovic, Patrick Forterre

**Affiliations:** 1 Unité Biologie Moléculaire du Gène chez les Extrêmophiles, Department of Microbiology, Institut Pasteur, Paris, France; 2 Institut de Génétique et Microbiologie, CNRS-UMR 8621, Université Paris-Sud 11, Orsay, France; University of Kansas Medical Center, United States of America

## Abstract

The *Microviridae* comprises icosahedral lytic viruses with circular single-stranded DNA genomes. The family is divided into two distinct groups based on genome characteristics and virion structure. Viruses infecting enterobacteria belong to the genus *Microvirus*, whereas those infecting obligate parasitic bacteria, such as *Chlamydia*, *Spiroplasma* and *Bdellovibrio*, are classified into a subfamily, the *Gokushovirinae*. Recent metagenomic studies suggest that members of the *Microviridae* might also play an important role in marine environments. In this study we present the identification and characterization of *Microviridae*-related prophages integrated in the genomes of species of the *Bacteroidetes*, a phylum not previously known to be associated with microviruses. Searches against metagenomic databases revealed the presence of highly similar sequences in the human gut. This is the first report indicating that viruses of the *Microviridae* lysogenize their hosts. Absence of associated integrase-coding genes and apparent recombination with *dif*-like sequences suggests that *Bacteroidetes*-associated microviruses are likely to rely on the cellular chromosome dimer resolution machinery. Phylogenetic analysis of the putative major capsid proteins places the identified proviruses into a group separate from the previously characterized microviruses and gokushoviruses, suggesting that the genetic diversity and host range of bacteriophages in the family *Microviridae* is wider than currently appreciated.

## Introduction

A number of ecological studies have revealed that microbial viruses predominate in the biosphere and outnumber their hosts by at least one order of magnitude [1], [2]. Due to their abundance and consequent influence on the composition and diversity of microbial communities, viruses can be rightfully considered to be the “major players in the global ecosystem” [3], [4]. Until recently, the majority of viruses in the environment were believed to possess double-stranded DNA genomes [2]. However, technological advances in single-stranded (ss) DNA amplification and sequencing from environmental samples revealed that viruses with ssDNA genomes are more prevalent in both soil and marine environments than previously recognized [5]–[8]. This realization precipitated an interest amongst environmental virologists in the diversity and distribution of ssDNA bacterial viruses in nature [7], [9]. Among ssDNA viruses that are most often identified in the environment using metagenomic approach are those belonging to the family *Microviridae*. However, the host organisms have yet to be determined.

The *Microviridae* comprises small isometric icosahedral viruses with circular single-stranded DNA genomes [10]. The members of this family are further divided into two subgroups based on structural and genomic differences. Viruses infecting enterobacteria belong to a genus *Microvirus* and are typified by microvirus phiX174. The other subgroup consists of viruses infecting obligate parasitic bacteria, such as *Chlamydia*, *Bdellovibrio* and *Spiroplasma* [11]. These viruses are grouped into subfamily *Gokushovirinae* (genera *Chlamydiamicrovirus*, *Bdellomicrovirus* and *Spiromicrovirus*) (http://www.ictvonline.org). Virions of phiX174-like microviruses are composed of four structural proteins (major capsid protein F, major spike protein G, DNA-binding protein J and DNA pilot protein H) [12]. In contrast, only two structural proteins, homologues of phiX174 proteins F and H, were identified in mature virions of gokushoviruses [13]. Furthermore, virion assembly in phiX174-like microviruses proceeds with the aid of two scaffolding proteins, internal scaffolding protein B and external scaffolding protein D [14]. The latter one does not have an equivalent in gokushoviruses. Consequently, the genomes of gokushoviruses are slightly smaller than those of microviruses (4.5 kb versus 5.3–6.2 kb). Viruses from both groups replicate their genomes via a rolling-circle (RCR) mechanism and encode dedicated RCR initiation proteins. All characterized members of the *Microviridae* are strictly lytic, unable to lysogenize their hosts [10]. However, the attempt to induce viruses from marine *Synechococcus* strains isolated from the Gulf of Mexico resulted in the production of icosahedral non-tailed virus-like particles that contained ssDNA [15], although detailed characterization of the virus-like particles was not performed. Furthermore, genomes of *Chlamydophila caviae* (formerly *Chlamydia psittaci*) and *Chlamydia pneumoniae* contain gene fragments showing sequence similarity to genes of *Chlamydia*-infecting gokushoviruses [16]. These observations suggest that the *Microviridae* might include not only lytic but also temperate members, as is the case for all other families of bacterial DNA viruses that possess circular genomes or replicate their genomes via a circular intermediate.

Unexplored diversity and abundance of the *Microviridae* viruses in the environment fuelled our interest in this virus group. In order to obtain more information about these viruses we analyzed the genomic sequences available in public databases for the presence of proviruses related to *Microviridae*. The rationale behind this approach is that a provirus, defective or not, represents a molecular record that a cell has been in contact with a particular virus [17]. In this study we identified seven proviruses that are related to members of the *Microviridae*. The proviruses are integrated in the genomes of different species of the order *Bacteroidales* (phylum *Bacteroidetes*). The identified proviruses are only distantly related to the previously characterized microviruses and gokushoviruses and may represent a new group or subfamily within the *Microviridae*. Searches against metagenomic databases suggest that these new viruses might be associated with the human gut microbiota. Our results presented here extend the knowledge on the evolution, diversity and host range of microviruses.

## Results and Discussion

### Identification of *Microviridae*-related proviruses

Bacterial and archaeal DNA viruses are often capable of integrating their genomes into the host chromosome thereby becoming proviruses. Even though proviruses related to *Microviridae* have not been previously reported, we set out to verify this possibility by performing searches against genomic sequences available in public databases. The ability to build a virion is the major feature distinguishing viruses from other mobile genetic elements, such as plasmids and transposons [18]. Therefore, to identify *Microviridae*-related proviruses the iterative BLAST searches were seeded with the major capsid protein (MCP) sequences of selected microviruses and gokushoviruses. Such targeted searches have previously yielded valuable information on the diversity and evolution of other bacterial and archaeal viruses [19], [20].

Iterative searches seeded with the MCP sequences of phiX174-like microviruses (protein F) did not return hits to proteins other than the orthologues encoded by microviruses and gokushoviruses. However, when the MCP sequences of gokushoviruses (protein VP1) were used as queries, significant hits to seven proteins encoded in the genomes of six different species of the phylum *Bacteroidetes* were obtained (Table 1). Notably, whereas protein sequences encoded by *Bacteroidetes* were obtained during the initial search (i.e., first iteration), the MCP orthologues encoded by microviruses were retrieved only after further iterations. This suggests that the MCPs of gokushoviruses are closer to the group of *Bacteroidetes*-encoded proteins than they are to the MCPs of microviruses.

**Table 1:** Coordinates of the putative BMV proviruses.

Provirus	Host organism	Source^a^	Contig accession number	Coordinates	Size, bp^b^
BMV1	*Bacteroides* sp. 2_2_4	human; gastrointestinal tract	NZ_EQ973357	453860..460215	6356
BMV2	*Bacteroides eggerthii* DSM 20697	human; feces	NZ_ABVO01000042	11368..17543	∼6176
BMV3	*Bacteroides plebeius* DSM 17135	human; feces	NZ_ABQC02000012	89498..95972	6483
BMV4	*Prevotella* sp. oral taxon 317 str. F0108	human; subgingival oral biofilm	NZ_GG740072	687371..687398	6733
BMV5	*Prevotella buccalis* ATCC 35310	human; dental plaque	NZ_ADEG01000016	69569..75677	6109
BMV6	*Prevotella bergensis* DSM 17361	human; soft tissue abscess	NZ_ACKS01000073+ NZ_ACKS01000072	1..700+78383..83169	700+4787
BMV7	*Prevotella bergensis* DSM 17361	human; soft tissue abscess	NZ_ACKS01000036	22909..27491	>4660

a – information regarding the source of species isolation was obtained either from original publications or from corresponding genome sequencing project homepages at NCBI.

b – distance between the first and the last nucleotides of two *dif*-like sites flanking the proviral region.

### Analysis of the proviral regions

**Figure 1:**
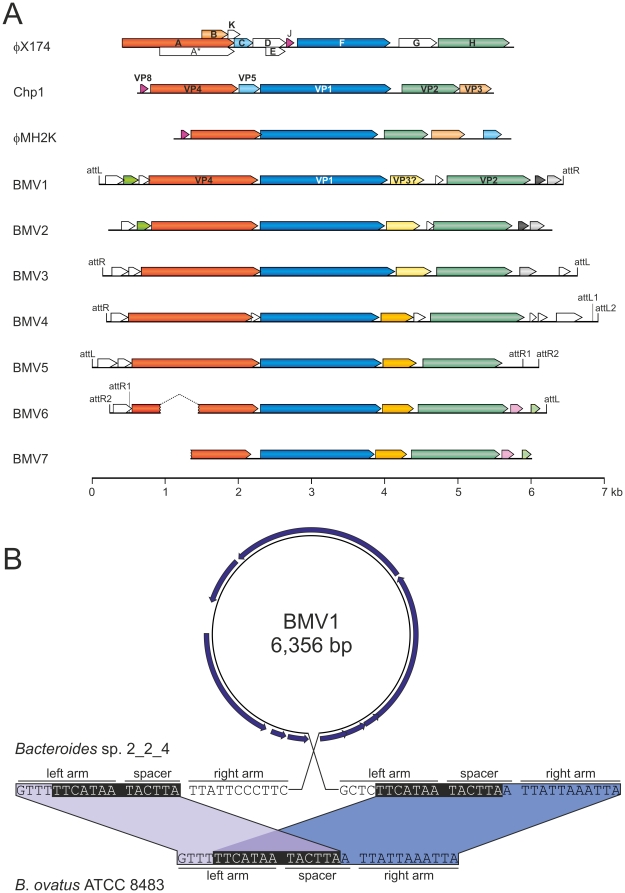
Bacteroidetes-associated, microvirus-related proviruses BMV1–7. A. Genomic organization of the putative BMV proviruses residing in the genomes of different species of the phylum *Bacteroidetes* and two gokushoviruses (family *Microviridae*), *Chlamydia* phage 1 (Chp1) and *Bdellovibrio*-infecting virus φMH2K. Circular genome maps of Chp1 (GenBank accession number: D00624) and φMH2K (GenBank accession number: AF306496) are linearized for convenient alignment. Open reading frames (ORF; arrows) are labeled according to the gokushovirus and microvirus protein nomenclature. ORFs encoding homologous products are coloured similarly. attL and attR, left and right attachment sites, respectively. B. Comparison of the BMV1-containing genomic region of *Bacteroides* sp. 2_2_4 with the provirus-free genomic region of *B*. *ovatus* ATCC 8483 (GenBank accession number: NZ_AAXF02000049; nucleotide coordinates: 301224–301252). The putative attachment sites flanking BMV1 as direct repeats are highlighted in black background.

In order to test whether the identified putative MCP-coding genes are of viral origin we performed a genomic context analysis. The regions of *Bacteroidetes* genomes adjacent to the MCP-coding genes were analysed for the presence of other viral genes. In all cases, immediately upstream of the *mcp* gene we identified a gene for an initiator of the rolling-circle replication (RCR) (Fig. 1A, Table S1). All three motifs characteristic to RCR proteins were found to be conserved (Fig. S1). Notably, as is the case for all known members of the *Microviridae*, motif III of the identified *Bacteroidetes* RCR proteins contains two invariable catalytic tyrosine residues (Fig. S1), a signature of superfamily I RCR proteins [21].

Transcriptionally downstream of the *mcp* genes we identified genes encoding homologues of the DNA pilot protein (protein H in microviruses or VP2 in gokushoviruses) (Fig. 1A, Table S1). The function of VP2/H-like proteins has been studied in the case of phiX174, but is yet to be fully understood [22]–[24]. Protein H is a multifunctional structural protein (12 copies per virion) required for piloting the viral DNA into the host cell interior during the entry process [10]. VP2 proteins of gokushoviruses share only limited primary structure similarity with H of microviruses [25]. However, VP2/H proteins from both groups of viruses share coiled-coil regions and predicted N-terminal transmembrane domains. Both these features are also characteristic to the VP2/H homologues (Table S1) encoded in the vicinity of the VP1/F-like *mcp* genes in the genomes of *Bacteroidetes*. Further sequence analysis did not reveal additional genes related to those of microviruses and/or gokushoviruses.

Mature virions of gokushoviruses are constructed of only two proteins, VP1 and VP2 [13]. Homologues of both proteins as well as the VP4-like RCR Rep protein are encoded as a block, within ∼6 kb region in the genomes of different species of *Bacteroidetes* (Fig. 1A). Furthermore, the organization of these genes is very similar to that found in the genomes of gokushoviruses (Fig. 1A). Consequently, these observations strongly suggest that this block of genes in *Bacteroidetes* genomes represents proviruses related to *Microviridae*. The seven proviral regions are refered to as BMV1–7, for *Bacteroidetes*-associated microviruses (Table 1).

Between the genes for VP1- and VP2-like proteins all BMVs contain open reading frames (ORFs) of approximately 150 codons (Fig. 1A, light and dark yellow arrows). Notably, despite being of similar size, ORFs from BMVs 1–3 (form *Bacteroides* species) share little sequence similarity (∼16 % identity at the protein level) with the corresponding ORFs from BMVs 4–7 (from *Prevotella* species) (Tables 1 and S1). The ORFs from either group have no homologues in public protein databases (except for those in BMVs). However, the conservation of the ORF within the two groups of BMVs suggested that it might encode an important function, possibly a scaffolding protein. To test this possibility, representative protein sequences from the two BMV groups were aligned with the sequence of VP3 scaffolding protein from φHM2K. The VP3 proteins of gokushoviruses are of ∼150 aa in length and share only limited sequence similarity with corresponding proteins from microviruses [25], [26]. Multiple sequence alignment revealed that proteins from the two BMV groups share a set of conserved residues not only with each other but also with the φHM2K VP3 (Fig. S2). We therefore predict that the conserved ORF following the one for the major capsid protein VP1 in all BMVs encodes a homologue of the internal scaffolding protein VP3.

Both microviruses and gokushoviruses encode very small (25–40 aa) DNA-binding proteins (protein J or VP8) that are rich in arginine and lysine residues [10]. The seven BMVs do not code for apparent homologues of J/VP8 proteins, nor were we able to identify homologues of any other proteins encoded by microviruses and gokushoviruses, including proteins C/VP5, D, G, or E.

### BMVs most likely rely on the host chromosome dimer resolution system for integration

The vast majority of temperate bacterial and archaeal viruses lysogenize their hosts by site-specifically integrating into the cellular chromosome. The reaction is generally catalyzed by a virus-encoded recombinase [27]. Interestingly, none of the BMVs encodes a recognizable recombinase, suggesting that the mechanism of integration is different from that utilized by the majority of prokaryotic viruses. Filamentous ssDNA viruses (family *Inoviridae*) infecting *Vibrio* species are an exception to this rule [28]. Filamentous vibrioviruses do not encode a recombinase of their own, but rather highjack the chromosome dimer resolution system of their hosts. Cellular tyrosine recombinases XerC and XerD recognize the *dif*-like sequences within the viral genome [29] and promote the integration of either the single-stranded (e.g., CTXφ; [30]) or the replicative, double-stranded (e.g., VGJφ; [31]) form of the viral genomic DNA into the chromosome dimer resolution sites.

In order to define the precise integration sites of BMVs, we took advantage of the availability of the genomic sequence for *Bacteroides ovatus* ATCC 8483, a provirus-free species closely related to BMV1-harboring *Bacteroides* sp. 2_2_4. Comparison of the corresponding sequences from the two *Bacteroides* species revealed the exact attachment site on the bacterial chromosome (Fig. 1B). It appears that BMV1 was integrated in the intergenic region between the genes for DNA mismatch repair protein MutS (GI:237722019) and glycoside hydrolase (GI:237722028). Due to recombination, the attachment site (13 bp) has been duplicated to flank the integrated provirus as direct repeats (Fig. 1B). With the size of 6.3 kb (Table 1) BMV1 genome is larger than those of all currently described gokushoviruses (4.5 kb) and microviruses (5.3 – 6.2 kb).

**Figure 2:**
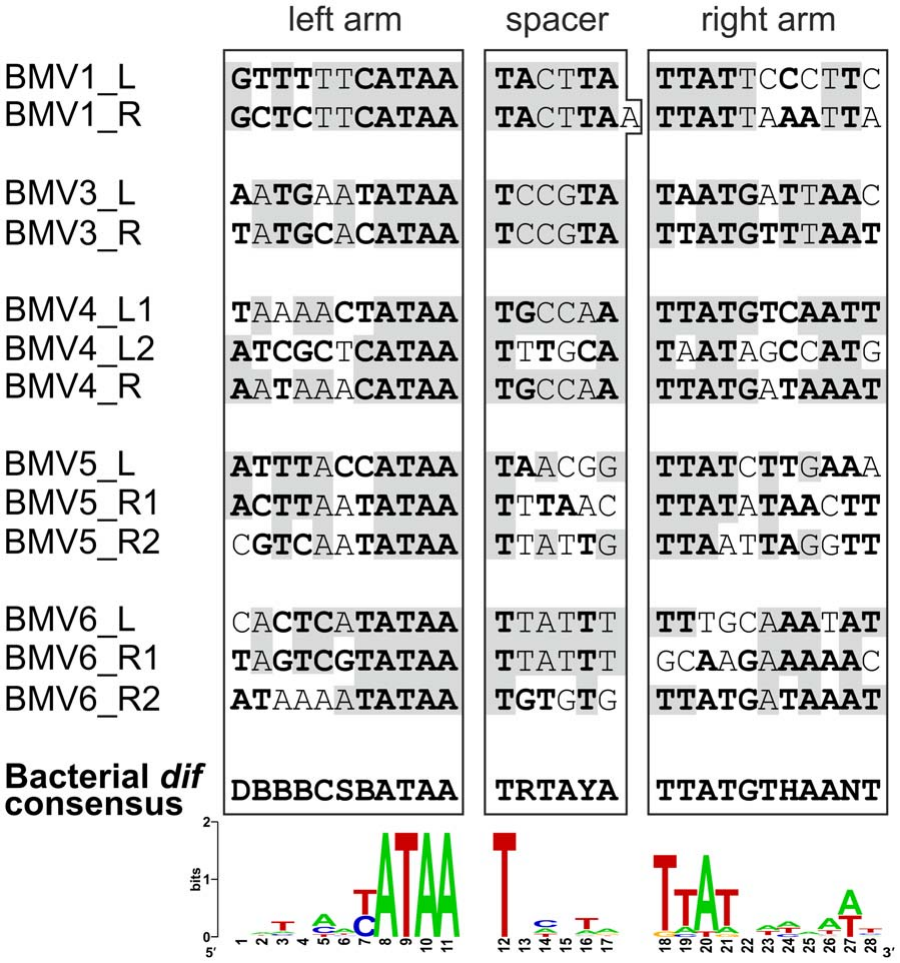
Comparison of the predicted attachment sites of BMVs with the bacterial *dif* consensus sequence. Left arm (XerC binding site), spacer and right arm (XerD binding site) regions of the *dif* sites are indicated. Bacterial *dif* consensus sequence [34] is indicated according to the IUPAC code. Nucleotide positions in the att sites of BMVs matching the *dif* consensus are shown in bold. Identical nucleotide positions in the left (L) and right (R) att sites, flanking the proviruses as imperfect direct repeats (see Figure 1), are shaded gray. BMV att consensus sequence is shown as sequence logo at the bottom of the figure.

Bacterial and archaeal *dif* sites are typically 28 bp long, display palindromic structure and are situated in intergenic regions, close to the GC-skew shift-point and replication terminus [32]–[34]. XerC/D recombinases act at the *dif* site to resolve chromosome dimers following replication termination [35], [36]. Careful examination of BMV1 revealed that sequences flanking the provirus resemble bacterial *dif* sites (Fig. 1B, 2), suggesting that, like in the case of filamentous vibrioviruses, the integration of BMV1 might have been mediated by the cellular recombination machinery. It should be noted that, genome sequences for all BMV-harbouring species are available as WGS genomic libraries (Table 1), precluding a meaningful GC-skew analysis of these bacterial chromsomes.

For the remaining six BMVs (BMV2–7), the integration sites could not be unequivocally defined by direct comparison of provirus-containing and provirus-free strains, due to unavailability of the genomic sequences for the latter group. We therefore investigated the proviruses (along with the flanking sequences) for the presence of *dif*-like sequences, similar to those found in BMV1. Indeed, such sequences turned out not to be specific to BMV1, but could also be identified in BMV3–6 (Fig. 2), but not in BMV2 and BMV7. It should be noted, however, that BMV7 sequence is only partial, present on the extremity of a contig (NZ_ACKS01000036) and misses the 5′-distal region of the gene for the RCR Rep protein along with the upstream region, including the attachment site (Fig. 1A). Interestingly, for BMVs 4–6, an additional *dif*-like sequence was identified in each of these proviruses, close to one of the termini of the integrated viral genome (see Fig. 1A, 2). This is reminiscent of CTXφ-like vibrioviruses, where viral genomes have two different *dif*-like sequences in inverted orientations [29], allowing the single-stranded form of the genome to be recombined with the host chromosome [30].

Chromosome dimer resolution has not been studied in any member of the phylum *Bacteroidetes*. Therefore, to ascertain whether XerC/D system might potentially be involved in this process we searched for the homologues of the *Escherichia coli* genes *xerC* and *xerD* in the genomes of *Bacteroidetes* species for which genome sequences are available. Genes for the two proteins are present in both BMV-carrying (Table S2) and BMV-free *Bacteroidetes* species, suggesting that the two cellular recombinases might indeed be involved in the integration of viral genomes at the chromosomal dimer resolution sites.

### BMVs are associated with human gut and oral microbiota

BMV-containing species fall into two different genera within the order *Bacteroidales*, *Bacteroides* (hosts for BMV1–3) and *Prevotella* (hosts for BMV4–7). *Bacteroidales* are gram-negative anaerobic bacteria that inhabit a variety of environments including the gastrointestinal tracts of mammals, the oral cavity of humans, soil and fresh water [37]–[39]. The six *Bacteroidales* species that contain BMV proviruses were isolated from humans (Table 1) and their genomes were sequenced as part of the Human Microbiome Project by NIH Human Microbiome Consortium [40]. In humans, *Bacteroides* constitute the dominant part of gut microbiota, whereas *Prevotella* are part of the normal flora of the human mouth and vagina [41]. Both *Bacteroides* and *Prevotella* are opportunistic human pathogens. Notably, *Prevotella bergensis* DSM 17361, the host for BMV6 and BMV7, was isolated from soft-tissue abscesses [42].

**Figure 3:**
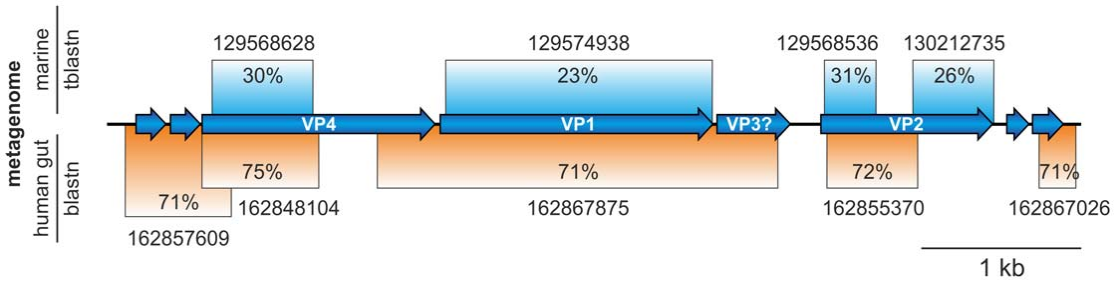
BMV-related sequences in environmental databases. Blastn (nucleotide query against nucleotide database) hits to the human gut metagenome and tblastn (protein query against translated nucleotide database) hits to marine GOS metagenome are depicted below and above the BMV2 genome map, respectively. Hit coverage and respective sequence identities (retrieved contigs are indicated by their GenBank identifiers) are also shown.

In order to get further insight into the distribution of BMV-like viruses in the environment, we searched the metagenomic databases at NCBI for the presence of sequences related to BMV proviruses. Searches seeded with the nucleotide sequence of BMV2 resulted in the most significant hits. These were to several contigs sequenced during the metagenomic analysis of the human gut microbiota [43]. The retrieved sequences were 71–75% identical (at the nucleotide level) to the BMV2 sequence and collectively covered ∼75% of the latter (Fig. 3). Notably, all five contigs matching the BMV2 provirus were generated by metagenomic sequencing of the faecal samples obtained from a single healthy male adult individual [43]. To identify more distantly related BMV-like sequences, further searches were performed against translated nucleotide sequences of the metagenomic databases using BMV2 protein sequences as queries. Sequences similar to the three conserved *Microviridae* proteins (VP1/F, VP2/H and VP4/A) of BMV2 were identified in the marine metagenome (Fig. 3), albeit the similarity was much lower (23–31% at the amino acid level) than to the human gut metagenome sequences. All marine samples containing BMV2-like sequences were collected during the Sorcerer II Global Ocean Sampling Expedition from surface marine waters along a voyage from Eastern North American coast to the Eastern Pacific Ocean [44]. It is not possible at the moment to tell whether the BMV-like sequences present in the metagenomic databases belong to free or integrated viruses. However, taking into account the information on the source of isolation of BMV-harbouring species and high sequence similarity to human gut-derived metagenomic sequences it is highly likely that BMV-like viruses are associated with human gut and oral microbiota.

### BMVs are closer to gokushoviruses but comprise a phylogenetically distinct group within *Microviridae*

**Figure 4:**
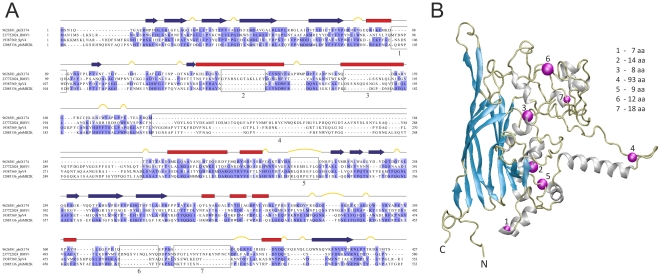
Analysis of the putative major capsid protein of BMV1. A. Alignment of the BMV1 major capsid protein sequence to the corresponding protein sequences of φX174, SpV4 and φMH2K. The proteins are denoted by their GenBank identifier followed by the corresponding (pro)virus name. The alignment is coloured according to sequence conservation (BLOSUM62 matrix). The secondary structure determined from the X-ray structure of φX174 capsid protein F (PDB ID: 1CD3) is shown above the alignment with α helices, β strands, and turns represented by red rectangles, blue arrows, and yellow bulges, respectively. Insertions (>5 aa) relative to the capsid protein F of φX174 are boxed. B. Atomic structure of the major capsid protein F of microvirus φX174 (PDB ID: 1CD3). Magenta spheres highlight the equivalent positions of the putative capsid protein of BMV1, where insertions (larger than 5 aa; numbered 1–7) occur relative to the capsid protein F of φX174 (refer to panel A for the alignment). Size of each insertion is indicated on the right of the figure.

Virions of gokushoviruses possess 'mushroom-like' protrusions positioned at the three-fold axes of symmetry of their icosahedral capsids. These structures are formed by large insertion loops within the MCP of gokushoviruses and are absent in the microviral MCPs [45]. In order to find out whether equivalent loops are also characteristic to BMV MCPs, a multiple sequence alignment of MCP sequences from BMVs, gokushoviruses and microviruses was constructed (Fig. S3). BMV1 MCP was found to be more closely related to corresponding proteins from gokushoviruses, sharing with the latter proteins six insertions (larger than 5 aa), including the one responsible for formation of 'mushroom-like' structures (insertion 4 in Fig. 4). Notably, the latter insertion in BMVs is considerably longer (93 aa in BMV1; Fig. 4) than in gokushoviruses and is accountable for the larger size of the BMV capsid proteins. In addition, the BMV1 MCP displayed a specific insertion of 14 aa (insertion 2 in Fig. 4), not present in the gokushoviral MCPs. All insertions were located outside of the predicted eight-stranded antiparallel beta-barrel core structure (Fig. 4). Therefore, it appears that not only genomic organization of BMVs is closer to that of gokushoviruses (Fig. 1A), but also their capsid proteins are more closely related.

**Figure 5:**
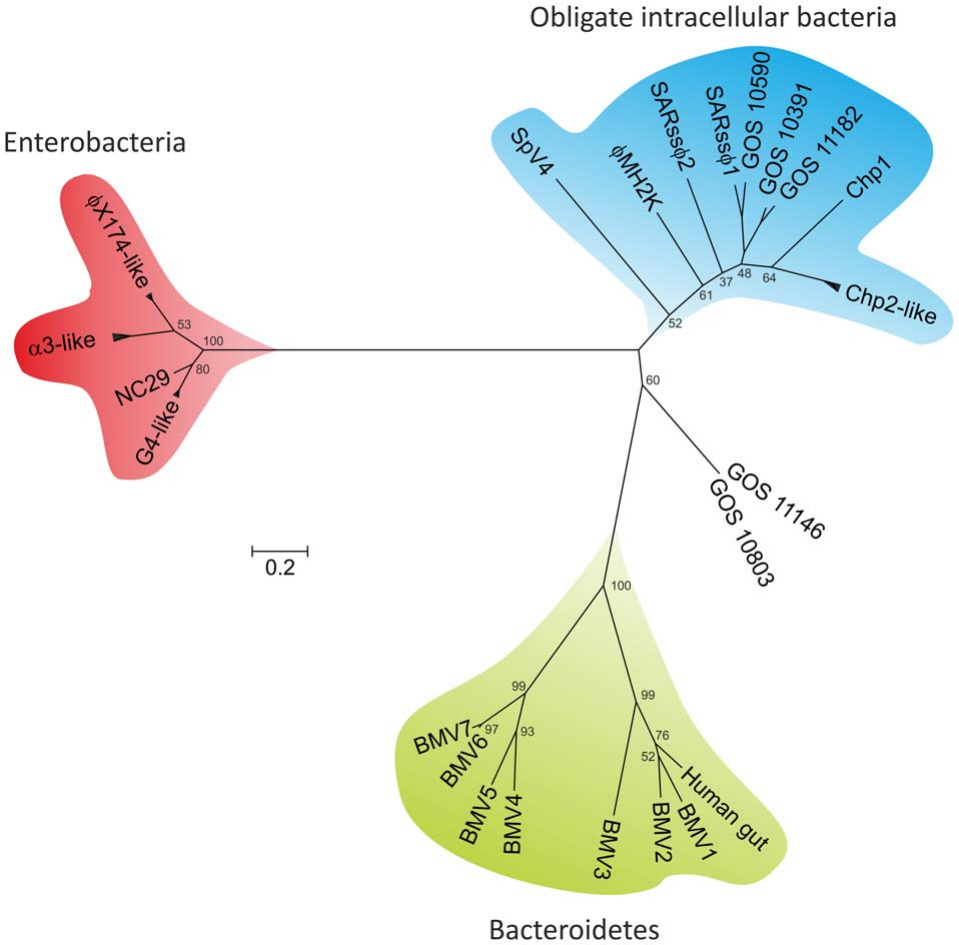
Unrooted phylogenetic tree of the φX174 F-like major capsid proteins. The evolutionary history was inferred by using the Maximum Likelihood method based on the Whelan and Goldman model [58]. The bootstrap consensus tree was inferred from 1000 replicates. Branches corresponding to partitions reproduced in less than 50% bootstrap replicates are collapsed. The scale bar represents the number of substitutions per site. The analysis involved 35 amino acid sequences. All positions containing gaps and missing data were eliminated. There were a total of 356 positions in the final dataset. φX174-like virus group includes φX174 (GI:9626381), WA10 (GI:71843040), S13 (GI:11095662), NC11 (GI:71842956), ID1 (GI:71842872). α3-like virus group includes α3 (GI:9625363), φK (GI:2493329), st-1 (GI:242346750). G4-like virus group includes G4 (GI:9626346), WA6 (GI:71843160), ID12 (GI:71843172). CPAR39-like virus group includes. Chp2-like virus group includes Chp2 (GI:9634949), Chp3 (GI:47566141), Chp4 (GI:77020115), CPAR39 (GI:9791178), φCPG1 (GI:17402851). SARssφ1 (GI:313766927) and SARssφ2 (GI:313766923) are uncultured microviruses the genomes of which were assembled by Tucker et al [9]. GOS sequences are microvirus-like major capsid proteins obtained during the Sorcerer II Global Ocean Sampling (GOS) Expedition [44]: GOS_10590 (GI:142008996), GOS_10391 (GI:142009231), GOS_11182 (GI:142008205), GOS_11146 (GI:142008257), GOS_10803 (GI:142008696).

Previous phylogenetic analysis of the MCP proteins supported the division of *Microviridae* viruses into two distinct groups, microviruses on one side and gokushoviruses on the other [26]. To better understand the relationship of BMVs to other members of the *Microviridae* family we performed a phylogenetic analysis of their major capsid proteins (Fig. 5). Our maximum likelihood analysis supported the previous conclusion regarding the relationship of microviruses and gokushoviruses [26] and revealed that BMVs fall into a third group, separate from the other two (Fig. 5). Within the BMV cluster there is a separation between the BMV1-like (BMV1–3 and the MCP sequence obtained from the human gut metagenome; Fig. 3) and BMV4-like proviruses (BMV4–7). Notably, the division of BMVs into two groups based on the MCP phylogeny is consistent with the genomic content analysis (Fig. 1A, Table S1).

Whereas the BMV MCPs (VP1) are closer to corresponding proteins from gokushoviruses, the VP2 proteins of BMVs are more similar to their homologues in microviruses (protein H), as judged from the BLAST analysis (Table S1). We therefore investigated the possibility of horizontal gene transfer (HGT) between the two groups of BMVs. For that we performed a phylogenetic analysis of the three proteins conserved in all BMVs, i.e., homologues of VP1/F, VP2/H and VP4/A. No signs of recent inter- or intra-group gene transfer events could be detected for the three analysed proteins, suggesting that HGT might be rare not only in lytic members of the *Microviridae* [10], [46], but also among the temperate BMV group.

### Concluding Remarks

Studies on various bacterial and archaeal proviruses have provided valuable information on the diversity, phylogenetic distribution and evolution of corresponding virus groups [19], [47]–[50]. Analysis of the putative proviruses described in the present study expands our knowledge on the viral family *Microviridae*. Not only is this the first time that members of the *Microviridae* are implicated in lysogenization of their hosts, but also the association of this virus group with *Bacteroidetes* has not been previously recognized. The host range of members of this virus family now covers four different bacterial phyla, namely *Proteobacteria* (microviruses and bdellomicrovirus), *Tenericutes* (spiromicrovirus), *Chlamydiae* (chlamydiamicroviruses), and *Bacteroidetes* (BMVs). Notably, BMVs are clearly distinct from the previously recognized microviruses and gokushoviruses. Consequently, if confirmed to produce genuine viruses, BMVs may represent a new group or subfamily within the *Microviridae*, which we propose to name *Alpavirinae* (*Alpa*: Sanskrit for 'small', 'minute'). BMVs appear to be associated with human gut and oral microbiota. In the future, it will be very interesting to explore the diversity of viruses infecting *Bacteroides* and *Prevotella*, to see what other new viral groups, in addition to BMVs, are associated with these bacteria. BMVs identified here are likely to integrate into the genomes of their hosts at the chromosome dimer resolution *dif* sites with the aid of cellular XerC/D recombination machinery, a route thought to be exclusively employed by filamentous vibrioviruses of the family *Inoviridae*. Studies on the integration of BMVs into the cellular chromosome therefore promise to provide further exciting insights on how bacterial viruses with small genomes highjack cellular machineries for their own needs.

## Methods

### Identification and analysis of proviral sequences

Putative microvirus-related prophages were identified by homology-based searches against the nonredundant protein database at NCBI. The major capsid protein (MCP) sequences of representative microviruses (phiX174, GI:9626381; α3, GI:9625363; G4, GI:9626346) and gokushoviruses (Chp1, GI:9629155; phiMH2K, GI:12085136; SpV4, GI:19387569) were used as queries in the PSI-BLAST searches [51] with the default parameters (BLOSUM62 matrix, 0.005 as an E-value cutoff). When MCP sequences of gokushoviruses were used as queries, in addition to homologues in other members of the *Microviridae*, significant hits were obtained (during the first or second iteration) to seven protein sequences encoded in the genomes of six different species belonging to the phylum *Bacteroidales*. The genomes of the six bacterial species (Table 1) were sequenced as part of the Human Microbiome Reference Genomes Project by NIH Human Microbiome Consortium [40]. Contigs encoding the MCP-like proteins were downloaded from NCBI and analysed for the presence of other viral proteins encoded in proximity of the *mcp* genes using CLC Genomics Workbench software package (CLC Bio, Inc.). Protein sequences of the identified proviruses are provided in Table S3.

Transmembrane domains were predicted using TMpred (http://www.ch.embnet.org/software/TMPRED_form.html) or TMHMM (http://www.cbs.dtu.dk/services/TMHMM/). Coiled-coil regions were identified using COILS (http://www.ch.embnet.org/software/COILS_form.html) [52]. Sequence logo was created with WebLogo (http://weblogo.berkeley.edu/) [53].

### Phylogenetic analysis

For phylogenetic analysis multiple sequence alignments were constructed using PROMALS3D [54] and MUSCLE [55], manually examined and edited. Sequence alignments were visualized using Jalview [56]. Maximum likelihood analysis was carried out using MEGA5 software [57] with a WAG amino acid substitution model [58]. The robustness of the trees was assessed by bootstrap analysis (1,000 replicates).

## Supporting Information

Figure S1 Alignment of the three conserved motifs (I–III) of superfamily I rolling circle replication proteins with corresponding motifs from the putative replication proteins of the BMV proviruses. The protein sequences are denoted by their GenBank identifiers followed by the corresponding (pro)virus name. The limits of the depicted motifs are indicated by the residue positions on each side of the alignment, with the total length of the protein given in parenthesis. The numbers within the alignment indicate the distance between the motifs.
(PDF)

Figure S2 Multiple alignment of the putative internal scaffolding proteins from BMVs 1 and 6 with the VP3 protein from *Bdellovibrio* gokushovirus φMH2K (GI:12085142).
(PDF)

Figure S3 Phylogenetic analysis of proteins conserved in BMV proviruses. The evolutionary history of the VP1/F-like, VP2/H-like and VP4/A-like proteins encoded by BMV proviruses was inferred by using the Maximum Likelihood method based on the Whelan and Goldman amino acid substitution model. Numbers at the branch-points represent bootstrap values (1000 replicates). The outgroups were chosen based on the BLAST analysis.
(PDF)

Table S1 Annotation of BMV1 and its comparison to BMV2–7.
(DOC)

Table S2 XerC and XerD homologues in organisms of *Bacteroidales* containing microvirus-related proviruses.
(DOC)

Table S3 BMV protein sequences.
(DOC)
