# Supplementary material for: Microviridae Goes Temperate: Microvirus-Related Proviruses Reside in the Genomes of Bacteroidetes
Source: PLoS One. 2011 May 10;6(5):e19893. doi: 10.1371/journal.pone.0019893 (PMC3091885; doi:10.1371/journal.pone.0019893)
Supplement: Figure S1 — Alignment of the three conserved motifs (I–III) of superfamily I rolling circle replication proteins with corresponding motifs from the putative replication proteins of the BMV proviruses. The protein sequences are denoted by their GenBank identifiers followed by the corresponding (pro)virus name. The limits of the depicted motifs are indicated by the residue positions on each side of the alignment, with the total length of the protein given in parenthesis. The numbers within the alignment indicate the distance between the motifs. (PDF) [file pone.0019893.s001.pdf]

		Motif I		Motif II		Motif III		
237722023_BMV1	57	FITLTY	94	FRPHYHLLL	32	CSNYVASYV	207	(497)
218131115_BMV2	58	FITLTY	94	FRPHYHILL	32	CSSYVAGYV	208	(499)
198274653_BMV3	56	FLTLTF	95	FRPHFHILL	62	CSSYVAGYV	237	(545)
288927737_BMV4	59	MFTLTY	108	LRPHYHGII	53	TAYYVAEYV	244	(569)
282877219_BMV5	61	FFTLTY	115	YRPHYHGIL	53	TAYYVASYV	253	(582)
9634955_Chlamydia microvirus Chp2	105	FLTLTY	44	QRPHYHLLI	42	SAGYVARYS	215	(336)
12085145_Bdellovibrio microvirus ϕMH2K	64	FLTLTY	50	KRPHWHVLI	43	SASYVARYA	181	(315)
19424731_Spiroplasma microvirus SpV4	75	FVTLTY	51	MRPHYHICF	43	SANYTARYT	193	(320)
9625356_Enterobacteria microvirus α3	183	FDTLTL	54	GRLHWHVVH	71	VAWYVTKYV	332	(345)
125661610_Enterobacteria microvirus ϕX174	199	FDTLTL	54	GRLHFHAVH	71	VGFYVAKYV	348	(346)
75093009_Xanthomonas inovirus Lf	158	FLTLTY	48	FRPHYHVML	29	CVGYLAKYA	259	(494)
45597172_Spiroplasma inovirus SVTS2	96	FLTLTY	49	GAVHFHIIL	30	VVKYLAKYI	199	(309)
74095490_Spiroplasma inovirus SVGII3	99	FTTLTY	47	GAVHFHIIF	30	VSKYVAKYV	200	(513)

Figure S1. Alignment of the three conserved motifs (I–III) of superfamily I rolling circle replication proteins with corresponding motifs from the putative replication proteins of the BMV proviruses. The protein sequences are denoted by their GenBank identifiers followed by the corresponding (pro)virus name. The limits of the depicted motifs are indicated by the residue positions on each side of the alignment, with the total length of the protein given in parenthesis. The numbers within the alignment indicate the distance between the motifs.
