# Supplementary material for: Microviridae Goes Temperate: Microvirus-Related Proviruses Reside in the Genomes of Bacteroidetes
Source: PLoS One. 2011 May 10;6(5):e19893. doi: 10.1371/journal.pone.0019893 (PMC3091885; doi:10.1371/journal.pone.0019893)
Supplement: Figure S3 — Phylogenetic analysis of proteins conserved in BMV proviruses. The evolutionary history of the VP1/F-like, VP2/H-like and VP4/A-like proteins encoded by BMV proviruses was inferred by using the Maximum Likelihood method based on the Whelan and Goldman amino acid substitution model. Numbers at the branch-points represent bootstrap values (1000 replicates). The outgroups were chosen based on the BLAST analysis. (PDF) [file pone.0019893.s003.pdf]

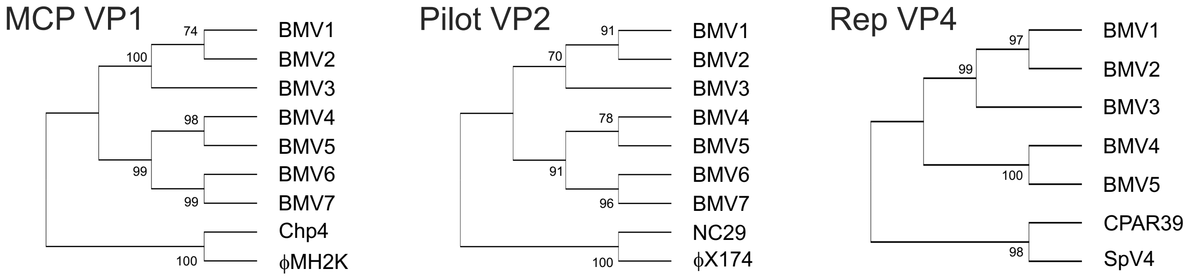

Figure S3. Phylogenetic analysis of proteins conserved in BMV proviruses. The evolutionary history of the VP1/F-like, VP2/H-like and VP4/A-like proteins encoded by BMV proviruses was inferred by using the Maximum Likelihood method based on the Whelan and Goldman amino acid substitution model. Numbers at the branch-points represent bootstrap values (1000 replicates). The outgroups were chosen based on the BLAST analysis.
