# Supplementary material for: Microviridae Goes Temperate: Microvirus-Related Proviruses Reside in the Genomes of Bacteroidetes
Source: PLoS One. 2011 May 10;6(5):e19893. doi: 10.1371/journal.pone.0019893 (PMC3091885; doi:10.1371/journal.pone.0019893)
Supplement: Table S1 — Annotation of BMV1 and its comparison to BMV2–7. (DOC) [file pone.0019893.s004.doc]

Table S1. Annotation of BMV1 and its comparison to BMV2–7.

| **Gene product** | **Function/feature** | **BMV1** | **BMV2** | **BMV3** | **BMV4** | **BMV5** | **BMV6** | **BMV7** | **Best hit outside BMV** |
| --- | --- | --- | --- | --- | --- | --- | --- | --- | --- |
| gp1 | Basic protein (17.2 % Arg + Lys) | ZP_04552501 |  |  |  |  |  |  |  |
| gp2 | 1TMD^b^ | ZP_04552502 | ZP_03459918; 27/56 (49%) |  |  |  |  |  |  |
| gp3 | 2TMD | ZP_04552503 |  |  |  |  |  |  |  |
| VP4^a^ | VP4/A-like RCR Rep; PFAM id: [PF05840](http://pfam.janelia.org/family?acc=PF05840) | ZP_04552504 | [ZP_03459919](http://www.ncbi.nlm.nih.gov/protein/218131115?report=genbank&log$=protalign&blast_rank=2&RID=FEU5AJ67011)^c^; 350/494 (71%) | [ZP_03207185](http://www.ncbi.nlm.nih.gov/protein/198274653?report=genbank&log$=protalign&blast_rank=3&RID=FEU5AJ67011); 178/555 (33%) | [ZP_06421584](http://www.ncbi.nlm.nih.gov/protein/288927737?report=genbank&log$=protalign&blast_rank=4&RID=FEUNVJ3M01S); 100/345 (29%) | [ZP_06286057](http://www.ncbi.nlm.nih.gov/protein/282877219?report=genbank&log$=protalign&blast_rank=5&RID=FEUNVJ3M01S); 88/347 (26%) | N-terminus: [ZP_06203524](http://www.ncbi.nlm.nih.gov/protein/270340023?report=genbank&log$=protalign&blast_rank=14&RID=FEUNVJ3M01S); 42/124 (34%)  Rest : ZP_06006772 | [ZP_06005936](http://www.ncbi.nlm.nih.gov/protein/261879509?report=genbank&log$=protalign&blast_rank=2&RID=FFAAH6VM01S); 260/275 (95%) identical to Rep of BMV6, but not BMV1 | *Chlamydia* phage CPAR39  ([NP_063900](http://www.ncbi.nlm.nih.gov/protein/9791179?report=genbank&log$=protalign&blast_rank=1&RID=FEWRTJJY011)): 51/198 (26%) – hit to BMV1 |
| VP1^a^ | VP1/F-like major capsid protein;  PFAM id: [PF02305](http://pfam.janelia.org/family?acc=PF02305) | ZP_04552505 | [ZP_03459920](http://www.ncbi.nlm.nih.gov/protein/218131116?report=genbank&log$=protalign&blast_rank=2&RID=FEUPM6P4016); 369/592 (63%) | [ZP_03207184](http://www.ncbi.nlm.nih.gov/protein/198274652?report=genbank&log$=protalign&blast_rank=3&RID=FEUPM6P4016); 281/619 (46%) | [ZP_06421583](http://www.ncbi.nlm.nih.gov/protein/288927736?report=genbank&log$=protalign&blast_rank=5&RID=FEUPM6P4016); 183/599 (31%) | [ZP_06286058](http://www.ncbi.nlm.nih.gov/protein/282877220?report=genbank&log$=protalign&blast_rank=7&RID=FEUPM6P4016); 177/595 (30%) | [ZP_06006771](http://www.ncbi.nlm.nih.gov/protein/270340022?report=genbank&log$=protalign&blast_rank=4&RID=FEUPM6P4016); 183/602 (31%) | [ZP_06005935](http://www.ncbi.nlm.nih.gov/protein/270339760?report=genbank&log$=protalign&blast_rank=6&RID=FEUPM6P4016); 175/576 (31%) | *Chlamydia* phage 4 ([YP_338238](http://www.ncbi.nlm.nih.gov/protein/77020115?report=genbank&log$=protalign&blast_rank=1&RID=FEYJGHTT014)): 140/554 (26%) – hit to BMV6 |
| VP3? |  | ZP_04552506 | [ZP_03459921](http://www.ncbi.nlm.nih.gov/protein/218131117?report=genbank&log$=protalign&blast_rank=2&RID=FEVMSKC6014); 83/135 (62%) | [ZP_03207183](http://www.ncbi.nlm.nih.gov/protein/198274651?report=genbank&log$=protalign&blast_rank=3&RID=FEVMSKC6014)^c^; 47/125 (38%) |  |  |  |  |  |
|  |  |  |  |  | [ZP_06421582](http://www.ncbi.nlm.nih.gov/protein/288927735?report=genbank&log$=protalign&blast_rank=2&RID=FF1D7HEF01S); 40/104 (39%) | [ZP_06286059](http://www.ncbi.nlm.nih.gov/protein/282877221?report=genbank&log$=protalign&blast_rank=1&RID=FF1D7HEF01S); 55/146 (38%) | NZ_ACKS01000072  (80211..80642) | NZ_ACKS01000072 (80211..80642);  142/144 (98%) |  |
| gp7 | Small basic protein (23.3 % Arg + Lys) | NZ_EQ973357 (458500..458589) ^d^ |  |  |  |  |  |  |  |
| VP2 ^a^ | VP2/H-like structural protein; coiled-coil regions, 1 TMD | ZP_04552507 | [ZP_03459922](http://www.ncbi.nlm.nih.gov/protein/218131118?report=genbank&log$=protalign&blast_rank=2&RID=FEVVDJ21016); 171/382 (45%) | [ZP_03207182](http://www.ncbi.nlm.nih.gov/protein/198274650?report=genbank&log$=protalign&blast_rank=3&RID=FEVVDJ21016); 98/355 (28%) | [ZP_06421580](http://www.ncbi.nlm.nih.gov/protein/288927733?report=genbank&log$=protalign&blast_rank=4&RID=FEVZYSEY01N); 55/260 (22%) | [ZP_06286060](http://www.ncbi.nlm.nih.gov/protein/282877222?report=genbank&log$=protalign&blast_rank=7&RID=FEVZYSEY01N); 47/271 (18%) | [ZP_06006770](http://www.ncbi.nlm.nih.gov/protein/261880343?report=genbank&log$=protalign&blast_rank=5&RID=FEVZYSEY01N); 58/300 (20%) | [ZP_06005934](http://www.ncbi.nlm.nih.gov/protein/261879507?report=genbank&log$=protalign&blast_rank=6&RID=FEVZYSEY01N); 56/315 (18%) | *Enterobacteria* phage NC29 ([AAZ49059](http://www.ncbi.nlm.nih.gov/protein/71842862?report=genbank&log$=protalign&blast_rank=1&RID=FEXP8G9401N)); 65/242 (27%) – hit to BMV2 |
| gp9 | 1TMD | NZ_EQ973357 (459812..459958) | NZ_ABVO01000042) (17077..17223); 23/48 (48%) |  |  |  |  |  |  |
| gp10 | 1TMD | ZP_04552508 | [ZP_03459923](http://www.ncbi.nlm.nih.gov/protein/218131119?report=genbank&log$=protalign&blast_rank=2&RID=FEW6RNBU016)^c^; 33/61 (55%) | NZ_ABQC02000012 (90058..90291); 19/63 (31%) |  |  |  |  |  |

a – putative proteins of BMVs that are related to proteins of microviruses or gokushoviruses are named according to the nomenclature adapted for chlamydiaviruses and phiMH2K.

b – TMD, transmembrane domain.

c – ORF was corrected based on BLAST results.

d – in the cases when an ORF has not been annotated in the original genome sequence, the accession number of the corresponding contig and exact coordinates of the ORF are provided.
