# Supplementary material for: Microviridae Goes Temperate: Microvirus-Related Proviruses Reside in the Genomes of Bacteroidetes
Source: PLoS One. 2011 May 10;6(5):e19893. doi: 10.1371/journal.pone.0019893 (PMC3091885; doi:10.1371/journal.pone.0019893)
Supplement: Table S2 — XerC and XerD homologues in organisms of Bacteroidales containing microvirus-related proviruses. (DOC) [file pone.0019893.s005.doc]

Table S2. XerC and XerD homologues in organisms of *Bacteroidales* containing microvirus-related proviruses.

| **Organism** | **XerC, GI** | **Similarity to *E*. *coli* homologue (GI: 16131663), sequence identity; E-value** | **XerD, GI** | **Similarity to *E*. *coli* homologue (GI: 16130796), sequence identity; E-value** |
| --- | --- | --- | --- | --- |
| *Bacteroides ovatus* ATCC 8483 | 160883082 | 102/291 (36%); 2e-44 | 160884146 | 112/291 (39%); 1e-51 |
| *Bacteroides* sp. 2_2_4 | 237718760 | 101/291 (35%); 3e-44 | 237721804 | 112/291 (39%); 6e-52 |
| *Bacteroides eggerthii* DSM 20697 | 218131354 | 98/296 (34%); 2e-42 | 160891605 | 108/291 (38%); 8e-52 |
| *Bacteroides plebeius* DSM 17135 | 198277427 | 102/288 (36%); 7e-33 | 198277388 | 110/285 (39%); 2e-50 |
| *Prevotella* sp. oral taxon 317 str. F0108 | 288929393 | 108/292 (37%); 5e-45 | 288928808 | 112/290 (39%); 8e-51 |
| *Prevotella buccalis* ATCC 35310 | 282878705 | 101/299 (34%); 9e-39 | 282879077 | 112/290 (39%); 3e-51 |
| *Prevotella bergensis* DSM 17361 | 261880904 | 106/296 (36%); 9e-40 | 261879004 | 116/295 (40%); 4e-53 |
