# Supplementary material for: Microviridae Goes Temperate: Microvirus-Related Proviruses Reside in the Genomes of Bacteroidetes
Source: PLoS One. 2011 May 10;6(5):e19893. doi: 10.1371/journal.pone.0019893 (PMC3091885; doi:10.1371/journal.pone.0019893)
Supplement: Table S3 — BMV protein sequences. (DOC) [file pone.0019893.s006.doc]

**Table S3.** BMV protein sequences.

BMV1 proteins

| gp1 | MKVREKYNIKKAFEAKCDWDFTIFMVMRYETIDGCTYRLKTPRLIPVHRFTLFAVTVIEASKIKKSINVLPQYVCTLTKIDENETDF |
| --- | --- |
| gp2 | MKLTSDQWNRIIQAIVTAVVTICNIILVSSCAVTMSMSVQKNNSSSTQQIEQKSESRNDSTTLDLSPNF |
| gp3 | MLWLLVILRFIVSVITVGILKVVKLALLRLFVLVVPVLMYLFFCNYGTKPLL |
| VP4^a^ | MVQNPFCKCLHPKRIMNPYTKESMVVPCGHCQACTLAKNSRYAFQCDLESYTAKHTLFITLTYANRFIPRAMFVDSIERPYGCDLIDKETGEILGPADLTEDERTNLLNKFYLFGDVPYLRKTDLQLFLKRLRYYVTKQKPSEKVRYFAVGEYGPVHFRPHYHLLLFLQSDEALQICSENISKAWTFGRVDCQVSKGQCSNYVASYVNSSCTIPKVFKASSVCPFSVHSQKLGQGFLDCQREKIYSLTPENFIRSSIVLNGKYKEFDVWRSCYSFFYPRCKGFVTKSSRERAYSYSIYDTARLLFPDAKTTFSLAKEIAIYIYYFHNPKETYLLDLYGYCSDQSKLYELSQYFYDSDVLLHSFNSGEFSRYVHRIYTELLISKHFLYFVCTHNTLAERKSKQRLIEEFYSRLDYMHLTKFFEAQQLFYESDLIGDDDLCTDNWDNSYYPYFYNNVYTDTNLFEKTPVYRLYSSDVKKLFNDRIKHKKLNDANKVFFE |
| VP1^a^ | MANIMSLKSLRNKTSRNGFDLSSKRNFTAKPGELLPVKCWEVLPGDKWSIDLKSFTRTQPLNTAAFARMREYYDFYFVPYNLLWNKANTVLTQMYDNPQHATSYIPSANQALAGVMPNVTCKGIADYLNLVAPDVTTTNSYEKNYFGYSRSLGTAKLLEYLGYGNFYTYATSKNNTWTKSPLSSNLQLNIYGVLAYQKIYADHIRDSQWEKVSPSCFNVDYLSGTVDSAMTIDSMITGQGFAPFYNMFDLRYCNWQKDLFHGVLPRQQYGDTAAVNVNLSNVLSAQYMVQTPDGDPVGGSPFSSTGVNLQTVNGSGTFTVLALRQAEFLQKWKEITQSGNKDYKDQIEKHWNVSVGEAYSEMSLYLGGTTASLDINEVVNNNITGSNAADIAGKGVVVGNGRISFDAGERYGLIMCIYHSLPLLDYTTDLVNPAFTKINSTDFAIPEFDRVGMESVPLVSLMNPLQSSYNVGSSILGYAPRYISYKTDVDSSVGAFKTTLKSWVMSYDNQSVINQLNYQDDPNNSPGTLVNYTNFKVNPNCVDPLFAVAASNSIDTDQFLCSSFFDVKVVRNLDTDGLPY |
| VP3? | MWQKRRIEPYVTPVRENIVGSKEMQCSEFREESPVDQFLFQEVSVDGDTSIRLSSDIYMLFNQQRLDKLSQTSLLEYFNNISVTEPRFNELRSKLGDEQLISFVKSRFIQSKSELMAWSNYLMNSTDEQIAALASAQSQQTQQSVQPVEPVSME |
| gp7 | MKKINANFLAEGNLDATSRCRPSGVRTKKV |
| VP2 | MASAAFIGIGSAIASAAISSAASAGTGVATTNAANAANKDIAQMNNEFNERMLQKQMDYNTLAYDQQVSDQWSFYNDAKQNAWDMFNATNEYNSASAQRERYEAAGLNPYVMMNTGSAGTAAATSATSATAPTKQGITPPTASPYSADYSGIMQGLGQAIDQLSSIPDKAKTIAETGNLKIEGKYKAAEAIARIANIKADTHSKKEQVALNKLMYSIQKDLASSTMAVNSQNIANMRAEEKFKNIQTLIADKQLSFMDATQKMELAEKAANIQLKLAQGALTRNQAAHEIKKISETEARTTLINEQTSLTIEQNTGQQLQNQAQRQQNRFDADTYNVRVKTLEESLFNIVFETDKLGAVKTVGKGIRAVGSVAKDIYDYFK |
| gp9 | MLFLLCSIFTIEYLNLFVLCLSKTLVIFPKGSRLIFSAILFLVLLYEK |
| gp10 | MNVYISGCPIPFHDLIEVFEYLRSLSPWLLYQYQFLDIVVNGVPRMYIMILYVNNVYNITYVCYH |

a – putative proteins of BMVs that are not related to proteins of microviruses or gokushoviruses are named according to the nomenclature adapted for chlamydiaviruses and phiMH2K.

BMV2

| gp1 | MRKSSSKRVSLKYRRVIIHFFSLYDGRVHRKIFKDCTLSEALAVFYAMSELHCWTCVDYYCIKY |
| --- | --- |
| gp2 | MDKEKIYKTIEIVVKAVVAIAAVWLCVSCTMSMSISKNNTNSNQSTEQSQATSVDSTTVDLNYK |
| VP4 | MSLFNPFCKCLNPQRIVNPYTNEAMTVPCGHCKACILAKNSRYAFQCDLESYSSKHTLFITLTYANRFIPRAQFVDSMERPYGHDLVDVETGEYLGEADLSIKEIERLQEKFHLFGYLPYLRKFDLQLFFKRFRYYVAKRFPKEKVRYFAIGEYGPVHFRPHYHILLFLQSDEALQVCSKVVSEAWPFGRVDCQLSKGKCSSYVAGYVNSSVLVPKVLTLPTLCPFCVHSQKLGQGFLQSERAKVYSLTPEQFVKRSIVINGRYKEFDVWRSAYAYFFPKCKGFADKSSRERAYSYGLYDTARRLFPSAETTFALAKEIVGYIYYFHNKKDTYCLDIFGEVSDQSDLYQFSQYFFEPEIVNYSLDSIEMCRYVHRVYTELLLSKHFLYFVCDRPTLSEQKRKLKLIEEFYSRLDYMHLKTFFENQQLFYESDLVGDLDLMSDAWENSYYPFFYDNVYFSSEVYKKTPVYRLYDMQISKLFSDRIKHKKLNDLNKIFVDE |
| VP1 | MANIMSLKSIRNKPSRNGFDLSFKKNFTAKAGELLPVMVKEVLPGDTFKINLKAFTRTQPVNTAAFARIREYYDFFFVPYDLLWNKANTVLTQMYDNPQHAVSIDPTRNFVLSGEMPYMTSEAIASYINALSTASALADYKSNYFGYNRSKSSVKLLEYLGYGNYESFLTDDWNTAPLMANLNHNIFGLLAYQKIYSDFYRDSQWERVSPSTFNVDYLDGSSMNLDNAYSTEFYQNYNFFDLRYCNWQKDLFHGVLPHQQYGETAVASITPDVTGKLTLSNFSTVGTSPTTASGTATKNLPAFDTVGDLSILVLRQAEFLQKWKEITQSGNKDYKDQLEKHWGVSVGDGFSELCTYLGGVSSSIDINEVINTNITGSAAADIAGKGVGVANGEINFNSNGRYGLIMCIYHCLPLLDYTTDMLDPAFLKVNSTDYAIPEFDRVGMQSMPLVQLMNPLRSFANASGLVLGYVPRYIDYKTSVDQSVGGFKRTLNSWVISYGNISVLKQVTLPNDAPPIEPSEPVPSVAPMNFTFFKVNPDCLDPIFAVQAGDDTNTDQFLCSSFFDIKAVRNLDTDGLPY |
| VP3? | MWCIKRRVEPYVDKFQPNNVNSAVLSGSEFVEPSPLHEFMFQEIECDGKKSIRITSDIYMLFNQQRLDKLTRSQLVEYFDNLSVSEPKMSDLRKKMTDEQLCSFVKSRFIQTPSELMAWSQYLMSSQDAMIAAAAVEIQPVSEPTPPPEPAPVAE |
| gp6 | MGGPLLRGGSRAMPYEIIPLKYKVMCNYKNIELWVLLL |
| VP2 | MGAAAMTGIVGSAIGAGTSLIGGASTTHMQNQANKEIAQMNNAFNEKMFDKQIAYNKEMYQTQLGDQWKFYDDQKANAWKLYEDNKAYQTEMWNKQNEYNDPSAQRARLEAAGLNPYMMMNGGSAGVAGSVSGTQGSAPSAGSPSAQGVQPPTATPYSADYSGVMQGLGHAIDTIMTGSQRNIQNAQADNLRIEGKYIASKAIAELYKTYNEAKNDDERVAIQRVLSSIQKDLSASQVAVNNENVRQIQAQTKIAVTENLLREQQLKFLPYEQRTQLALGAADIALKYAQKNLTEKQARHEIEKLAETIVRANGQAMQNQYDAETYRDRVKLVKESLFNAIYDTDKVGIFKTMSRAFSSFGVPDGVHK |
| gp8 | MVALCIIVYSRLFVIYLSKTLVIFLKGGRLMNISAVFFSCPIIRKMTV |
| gp9 | MNYSIQWCPIPFHDLMEIFDFLSSLSVVRLYQFDGLHILLNGFPIMQLIIAYVDGLYHITYRILRF |

BMV3

| gp1 | MRYSLVHRTRGKRIDFGSKSELIDWIKQHGFLLSDHVQRLWSLHRLRKTSDFFSSYVLSDRICSLFEYINLHKLLVK |
| --- | --- |
| gp2 | MAASCGSSWDFSGSNVTVTVCETDTVVSRGTILILPDTVKE |
| VP4 | MNYPFVSCLEPQRIKNKYTGEEMVVACKHCVACEQLRNFKYSNLCDFESLTAKKTVFLTLTFDDKFVPQFRFYKVGDDEYIMRDADTGEYLGRTLMTPQLMNEYQKRVNYRINYKGRFPYLSKRELQLFMKRLRKYLDKYEGQKIRFFATGEYGPLSFRPHFHILLFVDDPSLFLPSVHTLGEYPYPYWSKYQKAHCGKGTLLSKLEYYIRESWPFGGIDAQSVEQGSCSSYVAGYVNSSVPLPSCLKVDAVKSFSQHSRFLGRKIFGTELIPLLKLKFTEFVQRSFFCRGRYDNFRTPSEMLHSVYPQCKGFALLSHEQRFRVYTIWSRLRYYFNSDKKADVARSLVTSFYSWLDTGILRVPERVREDFLLIYTELSQNLNYKRIDRFDYDKFRHDDDLNNQLFQCVYSILLCSSVFEHNAKLWKSYLCSLSLMWCDFDRNELFLRKVERFWKNYEYLNLVDWYRKQEIYFDKSYSRKSDFLDGKERLSGDIKYFYNNVPYDVEQFKKRTFAYKAYSANVRFMARQRMKHKEQNDKNMIFETNI |
| VP1 | MKQIFDSMANIMSMKSVRNKPTRAGYDLTQKINFTAKAGSLIPVWWTPVLPFDDLNATVKSFVRTQPLNTAAFARMRGYFDFYFVPFRQMWNKFPTAITQMRTNLLHASGPVLADNVPLSDELPYFTAEQVADYIVSLADSKNQFGYYRAWLVCIILEYLGYGDFYPYIVEAAGGEGATWATRPMLNNLKFSPFPLFAYQKIYADFNRYTQWERSNPSTFNIDYISGSADSLQLDFTVEGFKDSFNLFDMRYSNWQRDLLHGTIPQAQYGEASAVPVSGSMQVVEGPTPPAFTTGQDGVAFLNGNVTIQGSSGYLQAQTSVGESRILRFNNTNSGLIVEGDSSFGVSILALRRAEAAQKWKEVALASEEDYPSQIEAHWGQSVNKAYSDMCQWLGSINIDLSINEVVNNNITGENAADIAGKGTMSGNGSINFNVGGQYGIVMCVFHVLPQLDYITSAPHFGTTLTNVLDFPIPEFDKIGMEQVPVIRGLNPVKPKDGDFKVSPNLYFGYAPQYYNWKTTLDKSMGEFRRSLKTWIIPFDDEALLAADSVDFPDNPNVEADSVKAGFFKVSPSVLDNLFAVKANSDLNTDQFLCSTLFDVNVVRSLDPNGLPY |
| VP3? | MAPLTSLRVPSPVEDFYREEVQTPLGSTPAVTYRNDIYMILNQRRLDSMTLAQFSDYLDHDRSASQLSQMREKMSDEQLHQFVKSRYIQHPSELRAWASYLDIEYSKEIQKLEEAVEAVKKQTSNTDPSPDPAPTPAVES |
| VP2 | MGKIAGAAIGAAGDVAAAGMQTTASIVNTNNTNKANKEIAESTNQANIQIAQMSNEYNREQLERQIEQEWDMWNAENEYNSASSQRKRLEEAGLNPYMMMDGGSAGSASSMTSPAAQPAVVPQMQGATMQPADMSGLSGLRGIASEFIATLKAQEDIRGQQLINEGQEIENQYKADKLLADLEKTRTESGFVRSQTKGQDIMNRFRPEMLSSEIRQRKTDTMFTQLRAHGQMLANLSAYQWYKVLPQQIKQTINEQMVRINNMKLQGNLTQAQINTEINKAVTEFMKGAREQQQFDFESDSYKDRLDQIKADLRHAIYNSGPEGAFGLFNVGQGFYDQIRGAFR |
| gp7 | MVEVSNFPMPFHDTDELCEFLLNCGKYLDWFDGQDVFVNGEYVLTIRVYWSQTHHCEYISYQTFGHPKSSRRRQNIK |
| gp8 | MLMYSLTPPKGWGDTEGFENMKSSHLSEGKSLNSTAISLYLGNTCANDTDTFG |

BMV4

| gp1 | MRRFQYYSYRLFLRYLLESDFELISMLPTSNGFVISYDDRYVSELDKLFGSLKTLVDFEVIEFVDGSTYDHKTRIEYQVL |
| --- | --- |
| VP4 | MSSDLKIFGNCLCPVHVHNRWTRDEMFVPCGRCEACVNAAASKQSKRVRNEIMQHKYSVMFTLTYNNEFIPRWERFLDNNDCPQLRPIGRCAELFPSCPLNYFDKVTGKWSIDLDTFLPKIENDEHTEVFASCCKKDIQNFLKRLRFNISKLYGKAESRKIRYYVASEYGPTTLRPHYHGIIFFDDASLLSEISSLIVRSWGFQRRVGGKRNSFIFQPFADISLTQQYVKLCDQNTAYYVAEYVSGNLGLPQVLAYKSTLPFHLCSKSPVIGCFKADYCEVLGRVHRGAYRVGREVFDEKSGQFMHYDIPLDRDLCSSLFRKCLGFSSLSFNEKLLRYSFYGQHFAEWYENANLAFIEWKYSTRLFSADFADFLRSERGWKYRSWLEVNYKYDYYYLEMDCDQTWYSSRNAWRVVRDFDMNCYLPWNDLYYTYVRLFDKFEVMRNSDQLIEFYKLFNDIVHECGFQQAMLAAYPLINDAVPVRTREKLLSECGMIYKEDAYLRSFFDEVAWFGDFYKFGILDYDKFSAYSFYRTQYFKNYLAQQQLRLLKRNKSKKLKNTFVFGSRQIS |
| gp3 | MYLVHVKFLDMRNIILSFFVGYGVGSLISHLCILFYSIIKFL |
| VP1 | MSLKKVPQIKPSRANRPRSAFDLSQKHLYTAPAGALLPVLSVDLMFHDHIRIQAQDFMRTMPMNSAAFISMRGVYEFFFVPYSQLWHPYDQFITSMNDYRSSVVSSAAGDKALDSVPNVKLADMYKFVRERTDKDIFGYPHSNNSCRLMDLLGYGKPITSSKTPVPLLYTGNVNLFRLLAYNKIYSDYYRNTTYEGVDVYSFNIDHKKGTFVPTADEFKKYLNLHYRNAPLDFYTNLRPTPLFTIGSDSFSSVLQLSDPTGSAGFSADGNSAKLNMASPDVLNVSAIRSAFALDKLLSISMRAGKTYAEQIEAHFGVTVSEGRDGQVYYLGGFDSNVQVGDVTQTSGTTNPNVSEVGNAKLAGYLGKITGKGTGSGYGEIQFDAKEPGVLMCIYSVVPAMQYDCMRLDPFVAKQTRGDYFIPEFENLGMQPIVPAFVSLNRAKDNSYGWQPRYSEYKTAFDINHGQFANGEPLSYWSIARARGSDTLNTFNVAALKINPHWLDSVFAVNYNGTEVTDCMFGYAHFNIEKVSDMTEDGMPRV |
| VP3? | MDIIDFYYPHLDSAQKKILLSETSPNTELPVLNAEISELQEVVFPTDPTTGLPINPVTKLLSGSITQLERDRILSFMQPMPSSKRNNLSDDDLIRMLPSRYNSTLVDMDAVRDWYEENIFQPLHEQELQQQQQQQQQQQQQQTGGQPVE |
| gp6 | MLVQFNFVSFLIQFIMKVFIKILKVLEIALPFLKTLVESLSKKEKKDNEDPTASV |
| VP2 | MFLFVYDLTGWVSAYSIQLAIIPALIGAGAAIVGGLLSRSGAKKANEQNYDFQREMWNKQVAQQDKVNAQQMAYQDKVNAENREWSTESNVRQRIEDAGYNPYLYNGQASANSTGMASSTNLGNSITPGSVTAVNEEEGLSNSLNSVGSIMAQGVKTAQDAYTLSRGKAIDKQNDKVSGIKGGTESAQAQATLQASQNEARIKASTAILQDMEVAILQTQAMDKNGQPMVDESTGRPVTLAMQRERGQQQEVMYRVEKLKSDILNGEVDWENTSVDTLLKRYQLEKTNPEQLAILRQTFSNLKDTNLQIKAETKVLSTQVGLNNSQTRLNIQNVATQKRYAKLLGQQTLTEEEKTLFQKLQNHYGSAEKVAEIVQKARPHNFIEFANYLIDTWHDDLTGRRTSYGKIAIDVDSIANNWKNRRKGMAFKK |
| gp8 | MLNAIPIAILIISFILSRFRIYANIVFSFYIFVNKD |
| gp9 | MCCETRRYLFLLQLTKITRHYKNRTILIHILTQNINIEPFVYIY |
| gp10 | MSVITNEEPNEVRRCSEVIANCGRRRDGLKLSVSEVHSASDHTKRTIRKAYVIVSRTLRVLPLPCLLLAKLTAHVVLHLLMIVCLLLSCARDLYARARKIVYAFCPLAGKKPRKSRVEYI |

BMV5

| gp1 | MRNLHPVAQSYYVAILRDETFIRCCVPVSRKAILAKSMKIEHFVNNALTIACNVARDEYATVLAYCPVDDSYYPLFNYNPFVKFISTPKR |
| --- | --- |
| gp2 | MFDMDPCAINSTPKYQNFTRCGSCGYAALYFDGSGHVAGLQCYFGSDDLTIVDLRQPDDMCDKV |
| VP4 | MIEPSHLKIVGNCLNPRKVYNPSLHGWMYCSCDKCTACLNQKATTLSNRARAEIEQHKYSVFFTLTYDNEHLPKYEVFQDSNEVIQYRPIGRLVDDSSSDMLSNSCPINKYNNYENLYQFDESTFIPPIENYEDIYHFGVVCKKDIQNFLKRLRWRISKIPNITKDESKIRYYISSEYGPTTYRPHYHGILFFDSKKILDKIKSLIVMSWGKYERQQGERNRFKFRPFARPSLTSDYIKLCDPNTAYYVASYVAGNDNLPQVLQLRETKPFHVQSKNPVIGSYKVDKQEILENINRGTYTTTKQVFDDKTGQFNDVIVPLPETTLSSIFRKCVCYSTLTNDVKQQLYSFYSKYLEEWKNHLTYEIYDLIVKENNLHALQVFGGFTSPDSLRVIYHYLHSYPYLKYRSFLRKYHSEEYNRLDMDTGQNWYASKNAFKQTKVLNFSKYYKHEDAIVSYLCLFDKYLLLRKMYTLKNFYELQDDLIKSVGMRSALLHCYPTMYEDIQTAKCKVKSYKFPESNNNVISYVQRYVSKHNLLMKPLNKTFEQSLYYKTFVNQQEQRLLKSTKQKKFNNSFINNQRLIY |
| VP1 | MSKKIPLIKASRANRPRNAFDLSQKHLFTAHAGMLLPVMTLDLIPHDHVSIQATDFMRCLPMNSAAFMSMRSVYEFFFVPYSQLWHPFDQFITGMNDYRSVLQSDLYKSKSPLVIPSFKRKELYELFNAPGGFLNQQSNQPDIFGFKSRFNFLRLLDLLGYGVYVNADGSSRIDAFSKLLDDTEKLSIFRLAAYQKIYSDFYRNTTYEAVDVSSFSLDNITDSISAINAFKRFGTLRYRNAQLDYFTNLRPTPLFDLDNPSLNSFYNTPGNADSVSIDSDSNAVNFQLDSDLLTVQSIRNAFALDKLMRITQRAGKTYAEQIKAHFGFEVSEGRDGRVNYIGGFDSNIQVGDVTQMSGTTASPEQGVSIKHGGYLGRVTGKAQGSGSGHIEFDAHEHGILMCIYSLVPDMQYDATRIDPFVTKLSRGDFFMPEFEDLGMQPLQTRYISDIRTQTEKFKGWQPRYSEYKTSLDINHGQFANGQPLSYWTVGRGRAGETLETFDIASLKINPKWLDSIFAVNYNGTQITDCVFGGCQFNVQKVSDMSENGEPRI |
| VP3? | MEKVNNSSQSIFTDIDVYMPHLSNEQKAAAFQVYVKPKNPSLCEDINKLADVLFPVDPVTGNPDNMVDKLISPNVNPMEKERILSFMQKMPASKRNNLSDKELMDMLPSRYNSTLTDIDKVRDFFENEIYTSLEDESKQEDSSASGTETSESTD |
| VP2 | MSPIIPFLEPAAGAFNGLLGAFGASRRQKRAIKNQWAMFEAQKQHQNEVNERAMAFQREMNQRNEDWNNESNVRQRVEDAGYNPYLYQNGAMSSNVNQSTPLAQNVAPTSPVDLPDPFSEFANSAIDSVKGIADILLTKQNTRNIAADSARAEYMLGRQKYKDSQKYAGYDLPTAEAQLTHTQLEDARQLMIKDTVEANLAIMTENISKSLAFDENDQPITDESGRQLMLKEVSDRSLLKSNEKLLDKIKAEIRRVEEESDLTKVKKKLEQFNLDNLAPAQVKEVYATIGRIESEIIQKKAAAEFIGEQVQTERVKRTGEIFSTGIKAKDYKYYEWNKLGQFGSKFIQLIPKRKGMAAKPKP |

BMV6

| gp1 | MEKTKDLSKAEMLMKSPRENPFQPRACRKRLICGQFDKVELHIEIYRAPKPNVLVFLDDLGRWLNFASLEDLFVFLANNYTTNI |
| --- | --- |
| VP4 | N-term:  MQVFSHCLNPKRIFNRYTKRFEYVACNECYACRNIRTSSMCHRVEQEIKSHRYSLFFTLTYNNDSLPRFEVFEDKKGCQQIRPCGRLASNYDRCPLVHSHRTEDPKLLIDSDVYAPYIQNDDCLNEFGVV  C-term:  KTKMLEDVYNGTVKYDSVSFKNNGEVSVTCVPYTKSDLCTVFRKCRGYRDLSAYAKSLIYSFVTNEYSQWKEKVTRLAQYDGFKSVKSFVRKNREYTLRHCLSSHPLYLALGMDDDSTWFASLYCHRMIEKYPLFSNIGFAHPVDAYLYLFGRFESLLELSLYHDFIDQLNDWYEWKGNKSIFAAYPFLSENIPVSFKDYKDLPLSYKNILHSTGIYIHLYDSNGVLNTSFIDDNLEVNSPEFVSFEIDSYKSFESRLKSKKANNKYFANYRKLD |
| VP1 | MSVSIPKIKATRPNRNRNAFDLSQRHLFTAHAGMLLPVLNLDLIPHDHVEINAQDFMRTLPMNTAAFASMRGVYEFFFVPYHQLWAQFDQFITGMNDFHSSANKSIQGGTSPLQVPYFNVDSVFNSLNTGKESGSGSTDDLQYKFKYGAFRLLDLLGYGRKFDSFGTAYPDNVSGLKNNLDYNCSVFRILAYNKIYQDYYRNSNYENFDTDSFNFDKFKGGLVDAKVVADLFKLRYRNAQTDYFTNLRQSQLFSFTTAFEDVDNINIAPRDYVKSDGSNFTRVNFGVDTDSSEGDFSVSSLRAAFAVDKLLSVTMRAGKTFQDQMRAHYGVEIPDSRDGRVNYLGGFDSDMQVSDVTQTSGTTATEYKPEAGYLGRVAGKGTGSGRGRIVFDAKEHGVLMCIYSLVPQIQYDCTRLDPMVDKLDRFDYFTPEFENLGMQPLNSSYISSFCTTDPKNPVLGYQPRYSEYKTALDVNHGQFAQSDALSSWSVSRFRRWTTFPQLEIADFKIDPGCLNSIFPVDYNGTEANDCVYGGCNFNIVKVSDMSVDGMPRV |
| VP3? | MELSIYFPHLSAAQLEVMSRRADYTSSGVCLNSKANEECDVICPYDEKLKRRPNVLSKVMDPNVNPLERERLMSTLQKVPVSKRNNLSDDELISMTPSRYNQTMTDDAAFASHLSHVVDDYSDSDSSASSSQGSDNSDSSNSDS |
| VP2 | MPLDPITGGALIAAGSSLLGGVLGAAGSSNLNRRNRRHQWDMMLQQQAYNDKVNQQQMDFQREVNQQNFAWNDPSNIRKRIEAAGYNPYLYDKQNLGSAQGSSLSSFSAGLGTPSELNPGAAFGEGIRGAASSFFDTLMKQKEIDAQTSRNDAFDYEMKKQVANDSLEDGGISAYYSSGLTAISQIQSAQAAAKINTVQSTFEQWRQEFYSRNAMDENGKPLVDENGEYVSNFDAEQQSNISRNVLAVEKLQQDILAGEVNIENMEIDQIVKRYDLEFVKPAELDNLKQSLAVMKSNIAANNASAQASLANAYASMQAGLSTNALRRYNVSAASWDAHAKKYNAKIAESQYNYEDPAAASKAWWLNNPVGFHLDPILGSLGQILGGARDAGIAYRTLKGSKPKNKPIKGFLR |
| gp6 | MDFEFFETEPEDFCRFRCIVMSGHGLPARFTWCSYRNIECPYLHNPTQCSDYEKPFY |
| gp7 | MPHGKPIGANISQKQPILPYYLSHFPPGVCPRQTSFSCFYF |

BMV7

| VP4 | C-term:  KTKMLEDVYNGTVKYDSVSFKNNGEVSVTRVPYTKSDLCAVFRKCRGYRDLSAYAKSLIYSFVTNEYPQWKEKVSRLAFYDGFKSVKSFVRKNREYTFRHCLSSHPLYLALGMDDDSTWFASLYCHRMIEKYPIFSNLGFAHPVDAYLYLFGRFESLLELSSYHDFIDQLNEWYEWKGNKSIFAAYPFLSENIPVSFKDYKDLSLSYKNILQSTGIYNHLYDSIGVLNTSFIDDNLEVNSPEFVSFEIDSYKSFQSRLKSKKANNKYFANYRKLD |
| --- | --- |
| VP1 | MLLPVLNLDLIPHDHVEINAQDFMRTLPMNTAAFASMRGVYEFFFVPYHQLWAQFDQFITGMNDFHSSANKSIQGGTSPLQVPYFNLESVFKNIIERDSTPSFQDDLQYRFKYGAFRLLDLLGYGRKFDSFGTAYPDNVSGLKNNLDYNCSVFRVLAYNKIYQDYYRNSNYENFDTDSFNFDKFKGGLVDAKVVADLFKLRYRNAQTDYFTNLRQSQLFTFIPEFSDDEHLNFDRDQYADQSKSNFTQLNFPVDVDNNLGYFSVSSLRSAFAVDKLLSVTMRAGKTFQDQMRAHYGVEIPDSRDGRVNYLGGFDSDLQVSDVTQTSGTTATEYKPEAGYLGRIAGKGTGSGRGRIVFDAKEHGVLMCIYSLVPQIQYDCTRLDPMVDKLDRFDFFTPEFENLGMQPLNSSYISSFCTPDPKNPVLGYQPRYSEYKTALDINHGQFAQNDALSSWSVSRFRRWTTFPQLEIADFKIDPGCLNSVFPVEFNGTESTDCVFGGCNFNIVKVSDMSVDGMPRV |
| VP3? | MELSIYFPHLSAAQLEVMSRRADYTSSGVCLNSKANEECDVICPYDDKLKRRPNVLSKVMDPNVNPLERERLMSTLQKVPVSKRNNLSDDELISMTPSRYNQTMTDDAAFASHLSHVVDDYSDSDSSATSSQGSDNSDSSNSDS |
| VP2 | MPLDPITGGALIAAGSSLLGGALGAAGSSNLNRRNRRHQWDMMLQQQAYNDKVNQQQMDFQREINQQNFAWNDPSNIRKRVEAAGYNPYLYDKQNLGSAQGVNLSSSSAGLGTPSEFNPGAALGEGIRGAATSFYDNFMKQQDIDARKKQIEVSDYEFEKQKKNDALTIGGISPYYSAGLSQIAQIQSAQAAAKINTIQSTFEQWRQEFYSRNAMDENGKPLVDENGEYVSNYDAEQQSNVTRNILGVEKLQQDMLAGKVNIESMEIDKLIKKYDLTFTKPQELENLKQSLSVMQSTIAANNASAQASLAAAYNQLMQGTTEEQSRVFKLGSLNWDRELKAQQHYSNDLNLRERKFNYDVQSEGRDLRGSWFGRYPGALFSTAGDWINRGLGPLLGPLSRIVVK |
| gp6 | MDFEFFETEPEDFCRFRCIVMSGHGLPARFTWCSYRNIECPYLHNPTQCSDYEKPFY |
| gp7 | MPHGKPIGANISQKQPILPYFLPHFPSGVCPRQTSFSCFYF |
