# Supplementary material for: Microviridae Goes Temperate: Microvirus-Related Proviruses Reside in the Genomes of Bacteroidetes
Source: PLoS One. 2011 May 10;6(5):e19893. doi: 10.1371/journal.pone.0019893 (PMC3091885; doi:10.1371/journal.pone.0019893)
Supplement: Figure S2 — Multiple alignment of the putative internal scaffolding proteins from BMVs 1 and 6 with the VP3 protein from Bdellovibrio gokushovirus φMH2K (GI:12085142). (PDF) [file pone.0019893.s002.pdf]

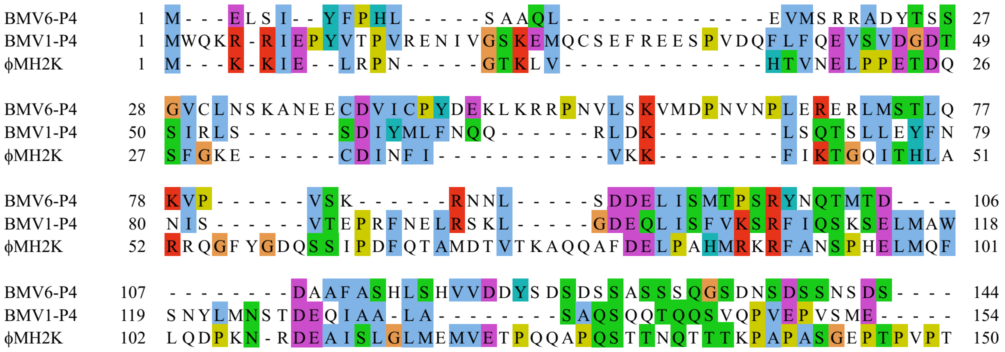

Figure S2. Multiple alignment of the putative internal scaffolding proteins from BMVs 1 and 6 with the VP3 protein from *Bdellovibrio* gokushovirus ϕMH2K (GI:12085142).
